# (7-Chloro-2-oxo-2*H*-chromen-4-yl)methyl di­ethyl­carbamodi­thio­ate

**DOI:** 10.1107/S160053681302240X

**Published:** 2013-08-14

**Authors:** T. G. Meenakshi, J. Shylajakumari, H. C. Devarajegowda, K. Mahesh Kumar, O. Kotresh

**Affiliations:** a Department of Physics, Y. Y. D. Govt. First Grade College, Belur 573 115, Hassan, Karnataka, India; b Department of Physics, AVK College for Women, Hassan 573 201, Karnataka, India; c Department of Physics, Yuvaraja’s College (Constituent College), University of Mysore, Mysore 570 005, Karnataka, India; d Department of Chemistry, Karnatak University’s Karnatak Science College, Dharwad, Karnataka 580 001, India

## Abstract

In the title compound, C_15_H_16_ClNO_2_S_2_, the 2*H*-chromene ring system is nearly planar, with a maximum deviation of 0.023 (2) Å. In the crystal, C—H⋯O hydrogen bonds give *R*_2_^1^(7) motifs, which generate [100] chains. C—H⋯π and π–π inter­actions between chromene moieties [shortest ring centroid–centroid distance = 3.6199 (13) Å] consolidate the packing.

## Related literature

For biological applications of coumarins and di­thio­carbamates, see: Abd Elhafez *et al.* (2003 ▶); Basanagouda *et al.* (2009 ▶); Borges *et al.* (2009 ▶); Bottomeley *et al.* (1985 ▶); Emmanuel-Giota *et al.* (2001 ▶); Hamdi & Dixneuf (2007 ▶); Marchenko *et al.* (2006 ▶); Teramoto *et al.* (1980 ▶); Trapkov *et al.* (1996 ▶). For a related structure and the synthesis of the title compound, see: Kumar *et al.* (2012 ▶).
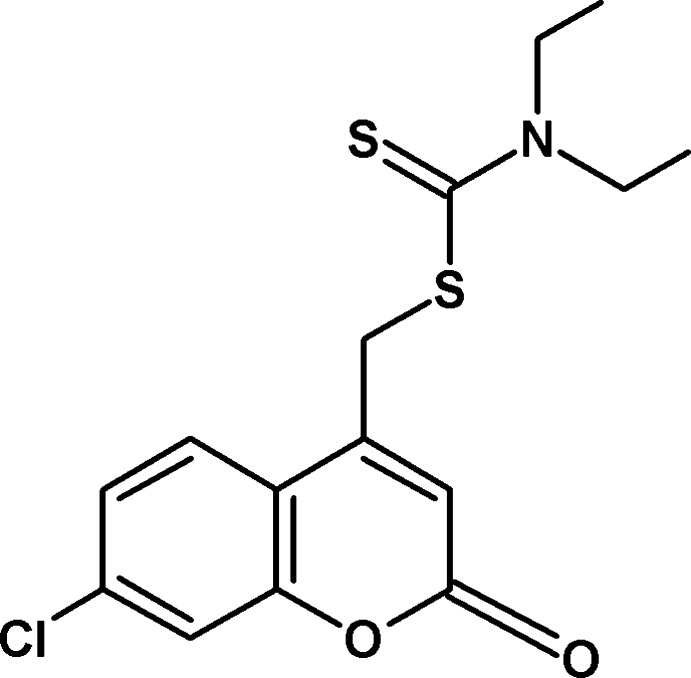


## Experimental

### 

#### Crystal data

C_15_H_16_ClNO_2_S_2_
*M* *_r_* = 341.86
Monoclinic, 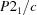

*a* = 7.7005 (2) Å
*b* = 23.3452 (8) Å
*c* = 9.7016 (3) Å
β = 110.349 (2)°
*V* = 1635.21 (9) Å^3^
*Z* = 4
Mo *K*α radiation
μ = 0.49 mm^−1^
*T* = 296 K
0.24 × 0.20 × 0.12 mm

#### Data collection

Bruker SMART CCD area-detector diffractometer
Absorption correction: multi-scan (*SADABS*; Sheldrick, 2007 ▶) *T*_min_ = 0.770, *T*_max_ = 1.000
11261 measured reflections
2865 independent reflections
2413 reflections with *I* > 2σ(*I*)
*R*_int_ = 0.024

#### Refinement

*R*[*F* ^2^ > 2σ(*F* ^2^)] = 0.039
*wR*(*F* ^2^) = 0.091
*S* = 1.08
2865 reflections
190 parameters
H-atom parameters constrained
Δρ_max_ = 0.38 e Å^−3^
Δρ_min_ = −0.28 e Å^−3^

### 

Data collection: *SMART* (Bruker, 2001 ▶); cell refinement: *SAINT* (Bruker, 2001 ▶); data reduction: *SAINT*; program(s) used to solve structure: *SHELXS97* (Sheldrick, 2008 ▶); program(s) used to refine structure: *SHELXL97* (Sheldrick, 2008 ▶); molecular graphics: *ORTEP-3 for Windows* (Farrugia, 2012 ▶); software used to prepare material for publication: *SHELXL97*.

## Supplementary Material

Crystal structure: contains datablock(s) I, global. DOI: 10.1107/S160053681302240X/zs2274sup1.cif

Structure factors: contains datablock(s) I. DOI: 10.1107/S160053681302240X/zs2274Isup2.hkl

Supplementary material file. DOI: 10.1107/S160053681302240X/zs2274Isup3.cml

Additional supplementary materials:  crystallographic information; 3D view; checkCIF report

## Figures and Tables

**Table 1:** Hydrogen-bond geometry (Å, °) *Cg*2 is the centroid of the C14/C15/C18–C21 ring.

*D*—H⋯*A*	*D*—H	H⋯*A*	*D*⋯*A*	*D*—H⋯*A*
C12—H12*A*⋯O5^i^	0.97	2.43	3.264 (3)	144
C18—H18⋯O5^i^	0.93	2.54	3.424 (3)	159
C8—H8*A*⋯*Cg*2^ii^	0.97	2.95	3.792 (3)	146

Symmetry codes: (i) 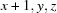; (ii) 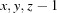.

**Fig. 1.**
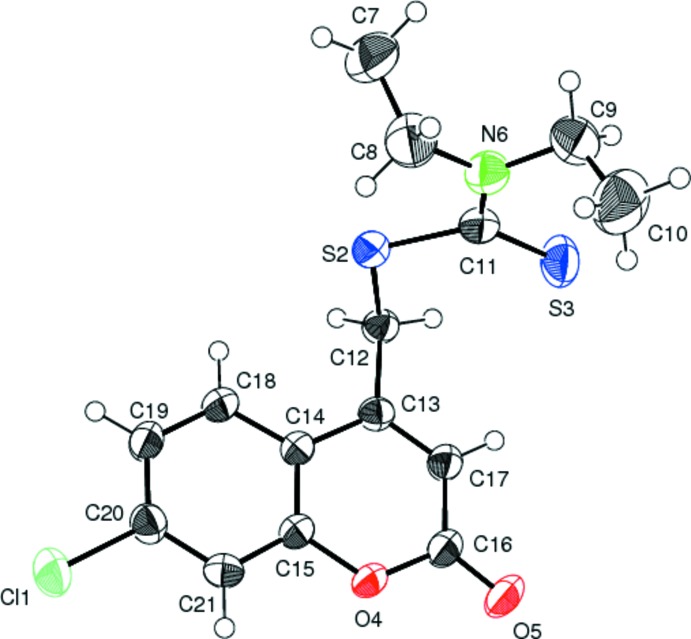
The molecular structure of the title compound. Displacement ellipsoids are drawn at the 50% probability level. Hydrogen atoms are shown as spheres of arbitrary radius.

**Fig. 2.**
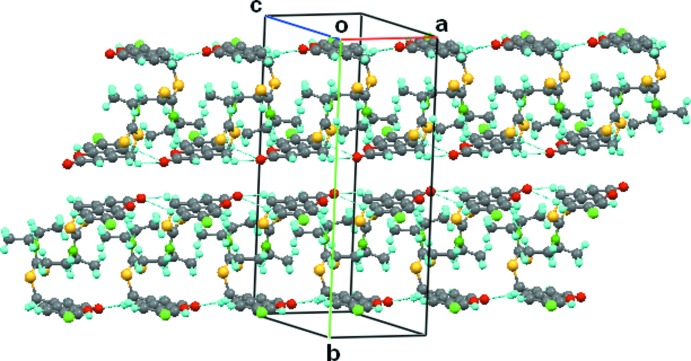
A perspective view of the packing of the molecules in the unit cell, with hydrogen bonds shown as dashed lines.

## supplementary crystallographic information

### 1. Comment

Coumarins are a large family of compounds, of natural and synthetic origin, that display a variety of pharmacological properties. Due to their structural variability they occupy an important place in the realm of natural products and synthetic organic chemistry. Recent studies pay special attention to their antioxidative and enzymatic inhibition properties (Borges *et al.*, 2009). Numerous functionalized coumarins have been presented as anti-bacterial (Abd Elhafez *et al.*, 2003; Basanagouda *et al.*, 2009), anti-oxidant (Trapkov *et al.*, 1996), anti-inflammatory (Emmanuel-Giota *et al.*, 2001; Hamdi & Dixneuf, 2007), anti-coagulant (Hamdi & Dixneuf, 2007) and anti-tumour (Marchenko *et al.*, 2006) agents.

Antifungal treatments are widely used to prevent the development of pathogenic fungi, which are responsible each year for various crop diseases leading to large economical losses. The dithiocarbamate fungicides form the most important class of pesticides for broad spectrum control of a variety of fungal diseases on seeds, fruits and vegetables. Among dithiocarbamates, ethylenebis (dithiocarbamates) are the most widely used fungicides in the world. Due to their non selectivity and multisite action they are still spread in large quantities in association with more specific pesticides. Despite their low acute toxicity, these fungicides constitute a pesticide family of environmental concern since many reports suspect them of inducing neurological troubles resembling Parkinson disease, carcinogenesis w and teratogenesis (Teramoto *et al.*, 1980; Bottomeley *et al.*, 1985). Therefore, the synthesis of new coumarin derivatives is of considerable interest. In order to study the influence of new substituents on the activity of the coumarin dithiocarbamates (Kumar *et al.*, 2012), the title compound, (7-chloro-2-oxo-2*H*-chromen-4-yl)methyl diethylcarbamodithioate, C_15_H_16_Cl N O_2_S_2_, has been synthesized and the structure is reported herein.

In this compound (Fig. 1), the 2*H*-chromene ring system is nearly planar, with a maximum deviation of 0.023 (2) Å. In the crystal, cyclic intermolecular C12—H12*A*···O5^i^ and C18—H18···O5^i^ hydrogen-bonding interactions (Table 1) through an *R*^1^_2_(7) ring motif, generate chains which extend along the *a* axis. In addition, C—H···π and π–π interactions involving the benzene ring of the chromene moiety defined by C14/C15/C18—C21) [shortest centroid–centroid distance = 3.6199 (13) Å] stabilize the crystal packing and give a two-dimensional layered structure lying along [0 1 0] (Fig. 2).

### 2. Experimental

All the chemicals used were of analytical reagent grade and were used directly without further purification. The title compound was synthesized according to the reported method (Kumar *et al.*, 2012). The compound was recrystallized from an ethanol-chloroform mixture giving colourless crystals (m.p. 383–385 K: yield, 88%).

### 3. Refinement

All H atoms were positioned geometrically, with C—H = 0.93 Å for aromatic H, C—H = 0.97 Å for methylene H and C—H = 0.96 Å for methyl H, and refined using a riding model with *U*_iso_(H) = 1.5*U*_eq_(C) for methyl H-atoms and *U*_iso_(H) = 1.2*U*_eq_(C) for all other H-atoms.

### Crystal data

**Table:**

C_15_H_16_ClNO_2_S_2_	*F*(000) = 712
*M**_r_* = 341.86	*D*_x_ = 1.389 Mg m^−^^3^
Monoclinic, *P*2_1_/*c*	Melting point = 383–385 K
Hall symbol: -P 2ybc	Mo *K*α radiation, λ = 0.71073 Å
*a* = 7.7005 (2) Å	Cell parameters from 2865 reflections
*b* = 23.3452 (8) Å	θ = 1.7–25.0°
*c* = 9.7016 (3) Å	µ = 0.49 mm^−^^1^
β = 110.349 (2)°	*T* = 296 K
*V* = 1635.21 (9) Å^3^	Plate, colourless
*Z* = 4	0.24 × 0.20 × 0.12 mm

### Data collection

**Table:**

Bruker SMART CCD area-detector diffractometer	2865 independent reflections
Radiation source: fine-focus sealed tube	2413 reflections with *I* > 2σ(*I*)
Graphite monochromator	*R*_int_ = 0.024
φ and ω scans	θ_max_ = 25.0°, θ_min_ = 1.7°
Absorption correction: multi-scan (*SADABS*; Sheldrick, 2007)	*h* = −9→9
*T*_min_ = 0.770, *T*_max_ = 1.000	*k* = −26→27
11261 measured reflections	*l* = −10→11

### Refinement

**Table:**

Refinement on *F*^2^	Primary atom site location: structure-invariant direct methods
Least-squares matrix: full	Secondary atom site location: difference Fourier map
*R*[*F*^2^ > 2σ(*F*^2^)] = 0.039	Hydrogen site location: inferred from neighbouring sites
*wR*(*F*^2^) = 0.091	H-atom parameters constrained
*S* = 1.08	*w* = 1/[σ^2^(*F*_o_^2^) + (0.0317*P*)^2^ + 1.0627*P*] where *P* = (*F*_o_^2^ + 2*F*_c_^2^)/3
2865 reflections	(Δ/σ)_max_ < 0.001
190 parameters	Δρ_max_ = 0.38 e Å^−^^3^
0 restraints	Δρ_min_ = −0.28 e Å^−^^3^

### Special details

**Table:**

Experimental. IR (KBr): 675 cm^-1^ (C—S), 1270 cm^-1^ (C=S), 1087 cm^-1^ (C—O), 856 cm^-1^ (C—N),1202 cm^-1^ (C—O—C), 1721 cm^-1^ (C=O). GCMS: m/e: 341. 1H NMR (400 MHz, CDCl_3_, \?,. p.p.m): 1.25–1.27 (m, 2H), 1.29–1.32(m, 2H, C_1_), 3.73–3.79 (m, 6H, C_17_), 4.04 (m, 2H, C_4_), 6.56 (s,1*H*, C_14_), 7.29 (m,1*H*, C_15_), 7.34 (s,1*H*, C_8_), 7.70(d, 1H). Mol. Formula: C_15_H_16_ClNO_2_S_2_. Elemental analysis for C_15_H_16_ClNO_2_S_2_: C, 52.65; H, 4.67; N, 4.03.
Geometry. All s.u.'s (except the s.u. in the dihedral angle between two l.s. planes) are estimated using the full covariance matrix. The cell s.u.'s are taken into account individually in the estimation of s.u.'s in distances, angles and torsion angles; correlations between s.u.'s in cell parameters are only used when they are defined by crystal symmetry. An approximate (isotropic) treatment of cell s.u.'s is used for estimating s.u.'s involving l.s. planes.
Refinement. Refinement of *F*^2^ against ALL reflections. The weighted *R*-factor *wR* and goodness of fit *S* are based on *F*^2^, conventional *R*-factors *R* are based on *F*, with *F* set to zero for negative *F*^2^. The threshold expression of *F*^2^ > 2σ(*F*^2^) is used only for calculating *R*-factors(gt) *etc*. and is not relevant to the choice of reflections for refinement. *R*-factors based on *F*^2^ are statistically about twice as large as those based on *F*, and *R*- factors based on ALL data will be even larger.

### Fractional atomic coordinates and isotropic or equivalent isotropic displacement parameters (Å^2^)

**Table:**

	*x*	*y*	*z*	*U*_iso_*/*U*_eq_	
Cl1	0.57033 (9)	0.42546 (3)	1.32189 (7)	0.0585 (2)	
S2	0.39037 (8)	0.35206 (3)	0.57232 (6)	0.04076 (17)	
S3	0.03698 (10)	0.35303 (3)	0.30671 (8)	0.0604 (2)	
O4	0.01217 (19)	0.44543 (7)	0.86479 (16)	0.0419 (4)	
O5	−0.2474 (2)	0.45502 (9)	0.6779 (2)	0.0622 (5)	
N6	0.2357 (3)	0.26131 (9)	0.4244 (2)	0.0508 (5)	
C7	0.5614 (4)	0.22830 (15)	0.4920 (4)	0.0815 (10)	
H7A	0.6573	0.2077	0.5657	0.122*	
H7B	0.6027	0.2666	0.4850	0.122*	
H7C	0.5334	0.2094	0.3989	0.122*	
C8	0.3895 (4)	0.23021 (12)	0.5337 (3)	0.0658 (8)	
H8A	0.3502	0.1914	0.5429	0.079*	
H8B	0.4192	0.2487	0.6286	0.079*	
C9	0.1075 (4)	0.22566 (13)	0.3077 (3)	0.0656 (8)	
H9A	0.1741	0.1929	0.2895	0.079*	
H9B	0.0612	0.2478	0.2176	0.079*	
C10	−0.0528 (5)	0.20496 (15)	0.3481 (5)	0.0893 (11)	
H10A	−0.1327	0.1819	0.2697	0.134*	
H10B	−0.1207	0.2372	0.3640	0.134*	
H10C	−0.0076	0.1825	0.4363	0.134*	
C11	0.2113 (3)	0.31763 (11)	0.4273 (3)	0.0411 (6)	
C12	0.3112 (3)	0.42508 (9)	0.5676 (2)	0.0357 (5)	
H12A	0.4168	0.4508	0.5943	0.043*	
H12B	0.2311	0.4345	0.4685	0.043*	
C13	0.2076 (3)	0.43346 (9)	0.6713 (2)	0.0321 (5)	
C14	0.3045 (3)	0.43248 (9)	0.8290 (2)	0.0315 (5)	
C15	0.2014 (3)	0.43771 (9)	0.9211 (2)	0.0335 (5)	
C16	−0.0825 (3)	0.44760 (11)	0.7160 (3)	0.0410 (6)	
C17	0.0229 (3)	0.44064 (10)	0.6207 (2)	0.0380 (5)	
H17	−0.0394	0.4411	0.5196	0.046*	
C18	0.4971 (3)	0.42649 (10)	0.8967 (2)	0.0365 (5)	
H18	0.5709	0.4239	0.8387	0.044*	
C19	0.5781 (3)	0.42443 (10)	1.0466 (2)	0.0392 (5)	
H19	0.7057	0.4203	1.0902	0.047*	
C20	0.4683 (3)	0.42857 (10)	1.1319 (2)	0.0384 (5)	
C21	0.2794 (3)	0.43545 (10)	1.0716 (2)	0.0384 (5)	
H21	0.2070	0.4385	1.1306	0.046*	

### Atomic displacement parameters (Å^2^)

**Table:**

	*U*^11^	*U*^22^	*U*^33^	*U*^12^	*U*^13^	*U*^23^
Cl1	0.0491 (4)	0.0892 (5)	0.0311 (3)	0.0021 (3)	0.0063 (3)	0.0033 (3)
S2	0.0367 (3)	0.0470 (4)	0.0369 (3)	0.0075 (3)	0.0107 (2)	−0.0014 (3)
S3	0.0507 (4)	0.0590 (4)	0.0541 (4)	0.0072 (3)	−0.0036 (3)	−0.0032 (3)
O4	0.0246 (7)	0.0666 (11)	0.0366 (9)	0.0023 (7)	0.0134 (7)	−0.0014 (8)
O5	0.0249 (8)	0.1095 (16)	0.0517 (11)	0.0071 (9)	0.0127 (8)	0.0029 (11)
N6	0.0462 (12)	0.0463 (13)	0.0573 (13)	0.0041 (10)	0.0150 (10)	−0.0056 (10)
C7	0.0573 (18)	0.078 (2)	0.100 (3)	0.0199 (16)	0.0165 (18)	−0.0167 (19)
C8	0.0703 (19)	0.0452 (16)	0.072 (2)	0.0111 (14)	0.0122 (16)	0.0004 (14)
C9	0.0649 (18)	0.0548 (17)	0.072 (2)	0.0003 (14)	0.0170 (16)	−0.0194 (15)
C10	0.067 (2)	0.076 (2)	0.120 (3)	−0.0166 (18)	0.027 (2)	−0.013 (2)
C11	0.0386 (12)	0.0494 (15)	0.0384 (13)	0.0022 (11)	0.0174 (11)	−0.0026 (11)
C12	0.0339 (11)	0.0416 (13)	0.0342 (12)	0.0008 (10)	0.0151 (10)	0.0022 (10)
C13	0.0325 (11)	0.0311 (11)	0.0346 (11)	−0.0011 (9)	0.0142 (9)	0.0001 (9)
C14	0.0279 (10)	0.0341 (12)	0.0326 (11)	−0.0010 (9)	0.0109 (9)	−0.0009 (9)
C15	0.0271 (10)	0.0389 (12)	0.0356 (12)	−0.0014 (9)	0.0121 (9)	−0.0009 (9)
C16	0.0274 (11)	0.0551 (15)	0.0398 (13)	0.0016 (10)	0.0107 (10)	0.0006 (11)
C17	0.0317 (11)	0.0497 (14)	0.0320 (12)	0.0021 (10)	0.0104 (9)	0.0018 (10)
C18	0.0292 (11)	0.0447 (13)	0.0380 (12)	0.0007 (9)	0.0145 (10)	−0.0008 (10)
C19	0.0281 (11)	0.0475 (14)	0.0388 (13)	0.0011 (10)	0.0077 (10)	0.0012 (11)
C20	0.0389 (12)	0.0424 (13)	0.0321 (12)	−0.0016 (10)	0.0102 (10)	0.0011 (10)
C21	0.0379 (12)	0.0470 (14)	0.0349 (12)	0.0001 (10)	0.0185 (10)	−0.0018 (10)

### Geometric parameters (Å, º)

**Table:**

Cl1—C20	1.735 (2)	C10—H10A	0.9600
S2—C11	1.783 (2)	C10—H10B	0.9600
S2—C12	1.806 (2)	C10—H10C	0.9600
S3—C11	1.662 (2)	C12—C13	1.498 (3)
O4—C16	1.373 (3)	C12—H12A	0.9700
O4—C15	1.379 (2)	C12—H12B	0.9700
O5—C16	1.205 (3)	C13—C17	1.344 (3)
N6—C11	1.330 (3)	C13—C14	1.451 (3)
N6—C9	1.474 (3)	C14—C15	1.392 (3)
N6—C8	1.477 (3)	C14—C18	1.404 (3)
C7—C8	1.513 (4)	C15—C21	1.373 (3)
C7—H7A	0.9600	C16—C17	1.437 (3)
C7—H7B	0.9600	C17—H17	0.9300
C7—H7C	0.9600	C18—C19	1.370 (3)
C8—H8A	0.9700	C18—H18	0.9300
C8—H8B	0.9700	C19—C20	1.378 (3)
C9—C10	1.498 (5)	C19—H19	0.9300
C9—H9A	0.9700	C20—C21	1.375 (3)
C9—H9B	0.9700	C21—H21	0.9300
			
C11—S2—C12	104.12 (11)	C13—C12—H12A	109.4
C16—O4—C15	121.54 (17)	S2—C12—H12A	109.4
C11—N6—C9	120.8 (2)	C13—C12—H12B	109.4
C11—N6—C8	123.7 (2)	S2—C12—H12B	109.4
C9—N6—C8	115.4 (2)	H12A—C12—H12B	108.0
C8—C7—H7A	109.5	C17—C13—C14	118.6 (2)
C8—C7—H7B	109.5	C17—C13—C12	120.9 (2)
H7A—C7—H7B	109.5	C14—C13—C12	120.47 (18)
C8—C7—H7C	109.5	C15—C14—C18	117.00 (19)
H7A—C7—H7C	109.5	C15—C14—C13	118.45 (18)
H7B—C7—H7C	109.5	C18—C14—C13	124.5 (2)
N6—C8—C7	112.4 (3)	C21—C15—O4	115.93 (19)
N6—C8—H8A	109.1	C21—C15—C14	122.90 (19)
C7—C8—H8A	109.1	O4—C15—C14	121.17 (19)
N6—C8—H8B	109.1	O5—C16—O4	116.5 (2)
C7—C8—H8B	109.1	O5—C16—C17	126.2 (2)
H8A—C8—H8B	107.9	O4—C16—C17	117.40 (18)
N6—C9—C10	112.0 (3)	C13—C17—C16	122.8 (2)
N6—C9—H9A	109.2	C13—C17—H17	118.6
C10—C9—H9A	109.2	C16—C17—H17	118.6
N6—C9—H9B	109.2	C19—C18—C14	121.1 (2)
C10—C9—H9B	109.2	C19—C18—H18	119.4
H9A—C9—H9B	107.9	C14—C18—H18	119.4
C9—C10—H10A	109.5	C18—C19—C20	119.2 (2)
C9—C10—H10B	109.5	C18—C19—H19	120.4
H10A—C10—H10B	109.5	C20—C19—H19	120.4
C9—C10—H10C	109.5	C21—C20—C19	122.1 (2)
H10A—C10—H10C	109.5	C21—C20—Cl1	118.57 (18)
H10B—C10—H10C	109.5	C19—C20—Cl1	119.29 (17)
N6—C11—S3	124.23 (19)	C15—C21—C20	117.6 (2)
N6—C11—S2	112.71 (17)	C15—C21—H21	121.2
S3—C11—S2	123.04 (15)	C20—C21—H21	121.2
C13—C12—S2	111.11 (15)		
			
C11—N6—C8—C7	−87.5 (3)	C18—C14—C15—C21	−2.1 (3)
C9—N6—C8—C7	92.0 (3)	C13—C14—C15—C21	177.9 (2)
C11—N6—C9—C10	−89.0 (3)	C18—C14—C15—O4	178.11 (19)
C8—N6—C9—C10	91.4 (3)	C13—C14—C15—O4	−2.0 (3)
C9—N6—C11—S3	1.6 (4)	C15—O4—C16—O5	−179.3 (2)
C8—N6—C11—S3	−178.9 (2)	C15—O4—C16—C17	0.9 (3)
C9—N6—C11—S2	−176.8 (2)	C14—C13—C17—C16	0.2 (3)
C8—N6—C11—S2	2.7 (3)	C12—C13—C17—C16	178.6 (2)
C12—S2—C11—N6	−172.85 (18)	O5—C16—C17—C13	178.8 (3)
C12—S2—C11—S3	8.80 (18)	O4—C16—C17—C13	−1.4 (4)
C11—S2—C12—C13	93.46 (17)	C15—C14—C18—C19	1.7 (3)
S2—C12—C13—C17	−108.0 (2)	C13—C14—C18—C19	−178.2 (2)
S2—C12—C13—C14	70.4 (2)	C14—C18—C19—C20	−0.3 (3)
C17—C13—C14—C15	1.4 (3)	C18—C19—C20—C21	−0.8 (4)
C12—C13—C14—C15	−177.0 (2)	C18—C19—C20—Cl1	179.55 (18)
C17—C13—C14—C18	−178.6 (2)	O4—C15—C21—C20	−179.1 (2)
C12—C13—C14—C18	2.9 (3)	C14—C15—C21—C20	1.0 (3)
C16—O4—C15—C21	−179.1 (2)	C19—C20—C21—C15	0.5 (4)
C16—O4—C15—C14	0.8 (3)	Cl1—C20—C21—C15	−179.90 (17)

### Hydrogen-bond geometry (Å, º)

*Cg*2 is the centroid of the C14/C15/C18–C21 ring.

**Table:**

*D*—H···*A*	*D*—H	H···*A*	*D*···*A*	*D*—H···*A*
C12—H12*A*···O5^i^	0.97	2.43	3.264 (3)	144
C18—H18···O5^i^	0.93	2.54	3.424 (3)	159
C8—H8*A*···*Cg*2^ii^	0.97	2.95	3.792 (3)	146

Symmetry codes: (i) *x*+1, *y*, *z*; (ii) *x*, *y*, *z*−1.
